# Changes in infection control practice for coronavirus disease 2019 (COVID-19) between 2020 and 2021: A nationwide questionnaire survey in Japan

**DOI:** 10.1017/ash.2021.177

**Published:** 2021-07-22

**Authors:** Hitoshi Honda, Akane Takamatsu, Hiroki Saito, Koh Okamoto

**Affiliations:** 1 Division of Infectious Diseases, Tokyo Metropolitan Tama Medical Center, Musashidai, Fuchu, Tokyo, Japan; 2 Emergency and Critical Care Medicine, St Marianna University School of Medicine Yokohama City Seibu Hospital, Yasashi Asahi ward, Yokohama, Kanagawa, Japan; 3 Department of Infectious Diseases, The Univerisity of Tokyo Hospital, Hongo, Bunkyo-ku, Tokyo, Japan

## Abstract

The coronavirus disease 2019 (COVID-19) pandemic has influenced current infection control practices in the healthcare setting. We surveyed 74 hospitals in Japan regarding changes in their infection control practices or policies between 2020 and the present. We found that the current hospital infection control practices for COVID-19 are adequate.

Since its inception in early 2020, the coronavirus disease 2019 (COVID-19) pandemic has posed significant challenges to infection control practices in various regions around the world. COVID-19 among healthcare workers (HCWs) and hospitalized patients has led to substantial increases in morbidity and mortality. Meanwhile, several new guidelines on infection control practices focusing on severe acute respiratory coronavirus virus 2 (SARS-CoV-2) have been hurriedly issued by professional societies.^1,2^ Despite the importance of preventive measures against nosocomial transmission of SARS-CoV-2, the optimal SARS-CoV-2 infection control practices for acute-care hospitals are still moot. Some unresolved infection control–related issues remain, including what the indications for testing are, when patients should be quarantined, what constitutes appropriate personal protective equipment (PPE), when isolation precaution should be discontinued, what precautions should be taken in high flow nasal cannula (HFNC) use, and how infection control practices vary by institutions. We investigated the current infection control practices at the participating institutions and compare the most current infection control practices (February–April 2021) with those of February–April 2020.

## Methods

The current study was based on responses to a survey regarding infection control practices against SARS-CoV-2 which was sent to Japanese tertiary-care hospitals. The survey asked about hospital characteristics (eg, location, type of hospital, cumulative number of hospitalized patients with COVID-19 at each institution), individual-level practice (eg, PPE use, and actual infection control practices against SARS-CoV-2), and hospital-level practice [eg, precautions in HFNC use, and noninvasive positive pressure ventilation (NPPV) use] during February–April 2020 and February–April 2021.

A draft of the questionnaire was developed by the primary investigators (H.H. and A.T.) based on the current infection control guidelines as well as our own clinical experience of COVID-19 infection control practice.^1,2^ This survey was reviewed by the other primary investigators (K.O. and H.S.) for finalization. The questionnaire was then distributed to participants (either infection control nurses, physicians or other infection control personnel) at hospitals caring for COVID-19 patients with a snowball sampling. The survey was conducted using Google forms from April 19, 2021, through May 16, 2021.^3^ The institutional review board at Tokyo Metropolitan Tama Medical Center approved the project.

## Results

During the study period, 74 hospitals responded to the survey. Table 1 shows the details of each institution. Approximately 65% of the participating institutions were in the Kanto region, which includes the Tokyo metropolitan area, the epicenter of the COVID-19 pandemic in Japan. Many hospitals (48 of 74, 64.9%) dispatched physicians from various subspecialties, including surgery, to care for patients with COVID-19. Hospital infection control measures relied heavily on local Japanese and US guidelines.

**Table 1.** Characteristics of the Participating Institutions (N=74)

Characteristics	Frequency,
	No. (%)
**Geographical location**	
Northern region	2 (2.7)
Northeastern region	3 (4.1)
Kanto region (eg, Tokyo)	48 (64.9)
Chubu region	4 (5.4)
Kansai region	7 (9.5)
Chugoku-Shikoku region	2 (2.7)
Kyushu-Okinawa region	8 (10.8)
**Type of hospital**	
University hospital	20 (27.0)
Public hospital	24 (32.4)
Private hospital	21 (28.4)
Others	9 (12.2)
**No. of beds**	
<200	5 (6.8)
200–399	11 (14.9)
400–599	22 (29.7)
≥600	36 (48.6)
**Cumulative no. of patients with COVID-19 at the time of the questionnaire**	
<100	15 (20.3)
100–199	10 (13.5)
200–399	23 (31.1)
400–599	11 (14.9)
600–799	2 (2.7)
≥800	13 (17.6)
**Questionnaire respondents**	
Physicians engaging in COVID-19 infection control	42 (56.8)
Infection control nurses	26 (35.1)
Others	6 (8.1)
**Primary services responsible for COVID-19 care**	
1–2 primary care services (either general medicine, infectious diseases or pulmonary medicine)	20 (27.0)
>3 primary care services including department of general medicine and critical care, but not surgery	20 (27.0)
>3 primary care services including department of general medicine, critical care, and surgery	28 (37.8)
Others	6 (8.1)
**Information sources for deciding hospital infection control measures against COVID-19**	
Local guidelines (ie, JSIPC, MHLW, JAID)	72 (97.3)
Guidelines from US professional societies (ie, CDC, NIH, IDSA, SHEA)	63 (85.1)
WHO guideline	47 (63.5)
Other guidelines	27 (36.5)

Note. JSIPC, Japanese Society for Infection Prevention and Control; MHLW, Ministry of Health, Labour and Welfare; JAID, The Japanese Association of Infectious Diseases; CDC, Centers for Disease Control and Prevention; NIH, The National Institutes of Health; IDSA, Infectious Diseases Society of America; SHEA, Society for Healthcare Epidemiology of America; WHO, World Health Organization.

For PPE use, the N95 mask only or in combination with a surgical mask with an integrated eye shield was commonly used in both 2020 and 2021. The usage rate for each type of PPE did not change significantly between 2020 and 2021. Hair covers were commonly used, whereas shoe covers were not (Table 2).

**Table 2.** Changes in COVID-19 Infection Control Practice Between 2020 and 2021 (N=74)

Infection Control Practice	Feb–Apr 2020,	Feb–Apr 2021,	Changes in Proportion, %
	No. (%)	No. (%)	
**Type of mask used for HCWs**			
N95 mask only	33 (44.6)	30 (40.5)	−4.1
N95 mask plus surgical mask with integrated eye-shield	27 (36.5)	28 (37.8)	+1.3
Surgical mask only	10 (13.5)	10 (13.5)	0
Other	4 (5.4)	6 (8.1)	+2.7
**Other PPE used in COVID-19 care (multiple answers allowed)**			
Hair cover	64 (86.5)	69 (93.2)	+6.7
Long-sleeved isolation gown	73 (98.6)	74 (100)	+1.4
Protective suits other than gown (apron or smock)	10 (13.5)	11 (14.9)	+1.4
Eye shield	60 (81.1)	58 (78.4)	−2.7
Face shield	51 (68.9)	56 (75.7)	+6.8
Shoe cover	8 (10.8)	7 (9.5)	−1.3
Single gloving	49 (66.2)	53 (71.6)	+5.4
Double gloving	40 (54.1)	36 (48.6)	−5.5
**Ultraviolet light device for terminal room decontamination**			
Yes	4 (5.4)	14 (18.9)	+13.5^a^
**N95 Mask sterilization by hydrogen peroxide gas**			
Yes	9 (12.3)	7 (9.5)	−2.8
**Type of room for patients with COVID-19 on designated ward (multiple answers allowed)**			
Private room with negative pressure function	46 (62.2)	52 (70.3)	+8.1
Private room without negative pressure function	53 (71.6)	54 (73.0)	+1.4
Multi-patient room with negative pressure function (cohorting)	18 (24.3)	25 (33.8)	+9.5
Multi-patient room without negative pressure function (cohorting)	28 (37.8)	35 (47.3)	+9.5
Other	8 (10.8)	3 (4.1)	−6.7
**Type of room for patients with COVID-19 in the ICU (multiple answers allowed)**			
Private ICU room with negative pressure function	43 (58.1)	49 (66.2)	+8.1
Private ICU room without negative pressure function	27 (36.5)	24 (32.4)	−4.1
Open-floored ICU room with negative pressure function (cohorting)	15 (20.3)	15 (20.3)	0
Open-floored ICU room without negative pressure function (cohorting)	4 (5.4)	8 (10.8)	+5.4
Other	11 (14.9)	12 (16.2)	+1.3
**Precaution for both patients with COVID-19 and HCW upon patient transfer**			
Patient: surgical mask only, HCW: surgical mask only	9 (12.2)	11 (14.9)	+2.7
Patient: surgical mask only, HCW: N95 mask only	3 (4.1)	5 (6.8)	+2.7
Patient: surgical mask only, HCW: contact and airborne protection	58 (78.4)	50 (67.6)	−10.8
Patient: N95 mask only, HCW: contact and airborne protection	0 (0)	3 (4.1)	+4.1
Others	4 (5.4)	5 (6.8)	+1.4
**In-hospital infection control protocol for HFNC use**			
HFNC only allowed in private rooms with negative pressure function	19 (25.7)	37 (50.0)	+24.3^a^
HFNC allowed in private rooms without negative pressure function	2 (1.4)	9 (12.2)	+10.8
HFNC allowed in COVID-19–designated multipatient rooms	3 (3.1)	9 (12.2)	+9.1
HFNC disallowed	38 (51.3)	11 (14.9)	−36.4^a^
No infection control protocol for HFNC use implemented	9 (12.2)	2 (2.7)	−9.5
Other	3 (4.1)	6 (8.1)	+4.0
**In-hospital infection control protocol for NPPV use**			
NPPV only allowed in private rooms with negative pressure function	14 (18.9)	27 (36.5)	+17.6^a^
NPPV allowed in private rooms without negative pressure function	1 (1.4)	4 (5.4)	+4.0
NPPV allowed in COVID-19–designated multipatient rooms	3 (4.1)	5 (6.8)	+2.7
NPPV disallowed	43 (58.1)	29 (39.2)	−18.9^a^
No infection control protocol for NPPV use implemented	9 (12.2)	4 (5.4)	−6.8
Other	4 (5.4)	5 (6.8)	+1.4
**Family meetings for patients with COVID-19 (noncritical) (multiple answers allowed except the response, “Visitors banned”)**			
Visitors banned from direct contact with patients	50 (67.6)	31 (41.9)	−25.7
Visitors allowed contact with patients via online video meetings	27 (36.5)	45 (60.8)	+24.3
Visitors allowed to enter room with full PPE	3 (4.1)	7 (9.5)	+5.4
Other	10 (13.5)	16 (21.6)	+8.1
**Family meetings for patients with COVID-19 (critical) (multiple answers allowed except the response “visitors banned”)**			
Visitors banned from direct contact with patients	28 (37.8)	13 (17.6)	−20.2
Visitors allowed contact with patients via online video meetings	24 (32.4)	39 (52.7)	+20.3
Visitors allowed to enter room with full PPE	16 (21.6)	26 (35.1)	+13.5
Other	24 (32.4)	25 (33.8)	+1.4
**Paper documentation/correspondence during the pandemic**			
Little (0%–10%) paper correspondence replaced by electronic medium	37 (50.0)	29 (39.2)	−10.8
Some (10%–50%) paper correspondence replaced by electronic medium	16 (21.6)	23 (31.1)	+9.5
Much (51%–90%) paper correspondence replaced by electronic medium	4 (5.4)	6 (8.1)	+2.7
Most (91%–100%) paper correspondence replaced by electronic medium	15 (20.3)	16 (21.6)	+1.3
Other	2 (2.7)	0 (0)	−2.7
**Rehabilitation services for patients with COVID-19**			
Physical therapy continuously provided	29 (39.2)	53 (71.6)	+32.4^a^
Occupational therapy continuously provided	19 (25.7)	37 (50.0)	+24.3^a^
Speech therapy continuously provided	14 (18.9)	33 (44.6)	+25.7^a^
**COVID-19 screening for admitted patients**			
None	42 (56.8)	13 (17.6)	−39.2^a^
Specific patient population screening (eg, ICU admission)	23 (31.1)	28 (37.8)	+6.7
Universal screening for all admitted patients	9 (12.2)	33 (44.6)	+32.4^a^
**Collateral damage due to COVID-19 infection control practices (multiple answers allowed)**			
None	53 (71.6)	67 (90.5)	+18.9^a^
Restrictions in private room use for patients with MDROs	8 (10.8)	6 (8.1)	−2.7
Infection control practices against MDROs compromised due to PPE shortage	12 (16.2)	2 (2.7)	−13.5^a^
Admission of patients with MDROs curtailed	4 (5.4)	1 (1.4)	−4.0

Note. HCW, healthcare worker; PPE, personal protective equipment; ICU, intensive care unit; HFNC, high-flow nasal cannula; NPPV, noninvasive positive pressure ventilation; MDRO, multidrug-resistant organism.

a Indicated *P* < .05 by the χ^2^ test or Fisher exact test.

In-hospital infection prevention measures showed considerable variation in NPPV and HFNC use, patient–family meeting arrangements, and rehabilitation services. Many participating hospitals implemented guidelines or policies to promote care for patients with COVID-19 in 2021. Moreover, substantial increases in the use of NPPV and HFNC, as well as the promotion of physical and devoted care for patients with COVID-19, occurred in 2021.

## Discussion

The present survey compared the SARS-CoV-2 infection control practices at the beginning of the pandemic (February through April 2020) and 1 year later (February–April 2021). The results revealed no significant change at the individual level (eg, in terms of the type of PPE used) but indicated significant changes in hospital-level practices between the periods.

Although the mode of transmission of SARS-CoV-2, via either droplet or airborne route has been debated, the use of the N95 mask or its equivalent is now considered a standard precaution at many of the hospitals surveyed. Moreover, wearing full PPE, including a long-sleeved isolation gown and some form of eye protection, appeared to be commonplace, thanks to the recommendations of the current guidelines on PPE use.^1^ However, significant variation was observed in the routine use of hair covers and shoe covers, which theoretically protect healthcare workers’ hair, scalp, and shoes from contamination by droplets, aerosols, and fomites. Covering the hair and shoes might be important for full protection, given the ability of SARS-CoV-2 to survive for days on surfaces.^4^ However, none of the current guidelines specifically address the use of these covers in routine practice. Given the high transmissibility of the virus, the efficacy of these coverings in COVID-19 care warrant further investigation in a real clinical setting.

In 2021, with increasing use of HFNC and NPPV for patients with COVID-19, many hospitals implemented infection control protocols for their use. HFNC appears to be preferred presumably because of its ease of use and possible lower aerosol production.^5^ However, because either respiratory device can increase the risk of exposure to the virus and can contribute to its nosocomial spread, implementing a specific policy for HFNC and NPPV use is imperative from the infection control perspective.

Progress in infection control practices in matters related to COVID-19 patient care also occurred in 2021. Increased availability of physical and occupational therapy, introduction of ultraviolet light devices for terminal room decontamination, and newly implemented online video devices for family meetings to enable greater contact between patients hospitalized with COVID-19 and their relatives reflect significant advances in the quality of infection control practices and patient/family care as well as responses specific to the exigencies of treating large numbers of elderly patients with COVID-19 in Japan.

This study had some limitations. Although the questionnaire was addressed to hospitals in various areas of Japan, two-thirds of the participating centers were in Tokyo (Kanto), which may indicate sampling bias. The influence of variations in infection control practices on the incidence of nosocomial transmission of SARS-CoV-2 was not assessed. Since the survey was conducted between April and May 2021, the present study did not assess the impact of vaccinating HCW or the emergence of new variants (eg, B.1.617) on infection control practices.

In conclusion, whereas standard PPE use was already established in February–April 2020, hospital-level infection control practices against SARS-CoV-2 changed markedly between 2020 and 2021. Advances in hospital-level infection control measures were likely driven by the needs or demands of inpatient care. Although the ideal strategies for infection control practice are still moot, the findings of the present study ensure the current infection control practices and improved hospital safety culture for HCWs over the year.

## References

[1] Updated healthcare infection prevention and control recommendations in response to COVID-19 vaccination. Centers for Disease Control and Prevention website. https://www.cdc.gov/coronavirus/2019-ncov/hcp/infection-control-after-vaccination.html Accessed May 25, 2021.

[2] Country & technical guidance—coronavirus disease (COVID-19). World Health Organization website. https://www.who.int/emergencies/diseases/novel-coronavirus-2019/technical-guidance-publications. Accessed May 25, 2021.

[3] Google forms. https://workspace.google.com/products/forms/. Accessed May 25, 2021.

[4] Chin AWH , Chu JTS , Perera MRA , et al. Stability of SARS-CoV-2 in different environmental conditions. Lancet Microbe 2020;1:e10. 32835322 10.1016/S2666-5247(20)30003-3 PMC7214863

[5] Raoof S , Nava S , Carpati C , Hill NS. High-flow, noninvasive ventilation and awake (nonintubation) proning in patients with coronavirus disease 2019 with respiratory failure. Chest 2020;158:1992–2002 32681847 10.1016/j.chest.2020.07.013 PMC7362846

